# Human body projectiles implantation in victims of suicide bombings and implications for health and emergency care providers: the 7/7 experience

**DOI:** 10.1308/003588412X13171221591772

**Published:** 2012-07

**Authors:** HDL Patel, S Dryden, A Gupta, N Stewart

**Affiliations:** ^1^Barts and The London NHS Trust,UK; ^2^Metropolitan Police,UK; ^3^Oxford University Hospitals NHS Trust,UK

**Keywords:** Suicide, Bombing, Bony, Projectile, Implantation

## Abstract

**INTRODUCTION:**

On 7 July 2005 four suicide bombings occurred on the London transport systems. In some of the injured survivors, bone fragments were embedded as biological foreign bodies. The aim of this study was to revisit those individuals who had sustained human projectile implantation injuries as a result of the bomb blasts at all scenes, review the process of body parts mapping and DNA identification at the scene, detail the management of such injuries and highlight the protocols that have been put in place for protection against blood borne pathogens.

**METHODS:**

We retrospectively reviewed 12 instances of victims who sustained human body projectile implantation injuries. The Metropolitan Police and forensic scientists identified the human projectiles using DNA profiling and mapped these on the involved carriages and those found outside. All human projectiles included were greater than 3cm^2^.

**RESULTS:**

Twelve cases had human projectile implantation injuries. Of these, two died at the scene and ten were treated in hospital. Projectiles were mapped at three of the four bomb blast sites. Our findings show that victims within a 2m radius of the blast had human projectile injuries. Eight of the allogenic bony fragments that were identified in the survivors originated from the suicide bomber. All the victims with an open wound should have prophylaxis against hepatitis B and serum stored for appropriate action against HIV and hepatitis C infection.

**CONCLUSIONS:**

All victims following a suicide bombing should be assumed to have human body projectile implantation injuries with blood products or bony fragments. All immediate care providers should receive prophylaxis against hepatitis B virus and appropriate action should be taken against HIV and hepatitis C infection.

On the morning of 7 July 2005 four suicide bombers detonated improvised high explosive devices in a coordinated attack on three underground trains and a double decker bus in central London. The victims were treated at various London hospitals. However, more than 50% of those injured were treated at the Royal London Hospital, a busy level 1 trauma centre in East London. A total of 775 people were injured in the London explosions, of those 24 were critically injured. Fifty-six people including the four suicide bombers died. It was the largest mass casualty in the UK since the Second World War.

In 2007 the counterterrorism centre for worldwide incidents reported that over 14,000 terrorist attacks occurred throughout the world, resulting in over 44,000 injuries and more than 22,000 deaths, a marked increase from 2006.^1^ The wars in Iraq and Afghanistan have greatly increased morbidity and mortality, especially with the added targeting by suicide bombers on foot or being vehicle based. These attacks are often coordinated to inflict maximum damage. This form of injury in civilian practice presents us with significant management issues, especially in hospitals where staff are not experienced with this form of trauma.

The type of injuries sustained by victims is dependent on their distance from the device or bomber, the surrounding environment, the size of the explosives used and the intervening structures or bodies between the bomber and the victims. The London bombings were only the second known bombings after the Moscow bombing in 2004 in a completely confined environment such as an underground setting. Zukerman^2^ and Maynard *et al*^3^ classified blast injuries according to the physical effects they have on the body as a direct result of the released energy from the explosive device (Table 1).

**Table 1:** Blast injury classification

Class	Mechanism	Common pathology
Primary	Blast overpressure from high explosives. Interacts with air–soft tissue interface in the body.	Tympanic membrane rupture; pulmonary barotrauma; gastrointestinal tract rupture or haemorrhage; traumatic brain injury/concussion; traumatic amputation
Secondary	Fragments and debris from the blast or objects affected by the blast (eg human impact projectiles)	Penetrating injuries affecting any part of the body
Tertiary	The blast wave knocks over or throws victims, causing impact with surrounding solid objects and the floor.	Fractures/amputations of limbs; open/closed brain injury
Quaternary	Any other form of injury or exacerbation of existing disease caused by the blast	Burns; exacerbation of respiratory disease; angina, myocardial infarction; crush injuries

These injuries are classified into: primary blast injuries, which are soft tissue injuries caused by barotraumas from the initial blast wave; secondary blast injuries, caused by objects turned into projectiles by the blast wind that follows the blast wave (eg part of bomber or victim); tertiary blast injuries, as people are displaced by the wave into stationary objects such as walls; and quaternary injuries that include miscellaneous injuries such as burns, inhalational injuries and post-traumatic stress.

Some of those treated at hospital demonstrated a unique pattern of injury in the form of human projectile implantation as a direct result of the bomb blast. These projectiles could have originated from either the suicide bomber or from other victims in the vicinity of the device. In 2006 a case series was published of the victims treated at the Royal London Hospital who had suffered human projectile injuries from the London bombings.^4^ In our study, we revisit all the victims from the underground scene but in particular focus on the DNA identification and body part mapping that helped identify human projectile injuries, the management of such injuries and the protocols that have been put in place for both victims and healthcare workers for protection against blood borne pathogens. It is crucial that the many lessons learnt from these attacks are presented so that we are prepared for any future attacks.

## Methods

The body parts dispersed by the blast wind were mapped out using forensic studies including DNA identification and body part mapping. The identification used was DNA profiling^5^ of bodies, body parts and survivors. The bodies and body parts from fatalities were identified using primary criteria for disaster victim identification.^6^ DNA profiled bodies and body parts were matched to items that next of kin identified as being personal to the victim and contained DNA material (toothbrush, razor, hairbrush etc). All testing was carried out by a diverse team of forensic scientists working at a number of UK laboratories and results were collated by the Metropolitan Police Counter Terrorism Command investigation team.

The projectiles evaluated in the report were those greater than 3cm^2^ or smaller bony fragments if recovered from a victim. This was combined with witness statements as well as hospital and postmortem data. In the patients who were taken to hospital, penetrating wounds were x-rayed to identify any bony injury or projectile fragments. These were then surgically removed and analysed subsequently using DNA testing to identify whether they originated from the bombers or from the other victims.

Regarding body part mapping, the body part positions at the three underground scenes were mapped by the authors by reviewing all scene photography, which recorded each body part in situ and its removal. This was compared with the grid systems devised by each of the four scene forensic managers and the disaster victim identification records, which recorded the packaging and removal of each body part prior to DNA testing, linking each part to a particular individual. A body part reconciliation list was drawn up for each victim (fatal and non-fatal) who had sustained loss of bodily material.

Because there was not a set procedure for drawing up scene grids and grid references and due to the variations in the environment, they differed substantially across the four scenes. Only body parts that the authors could accurately position were included in the mapping.

**Figure 1:**
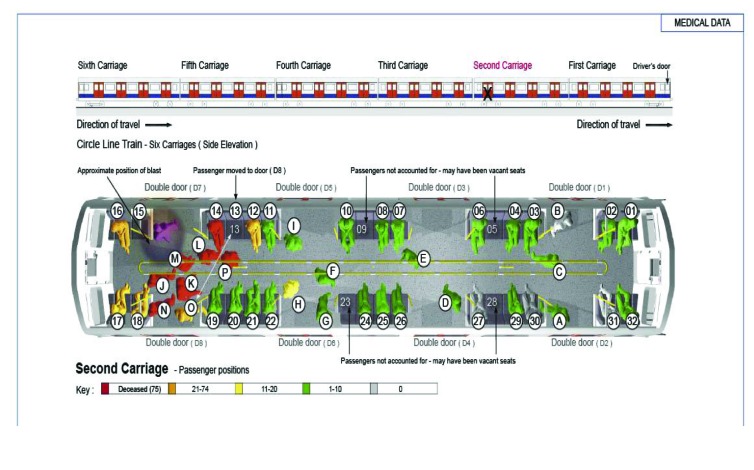
Pre-blast victim positional analysis at the Aldgate scene

**Figure 2:**
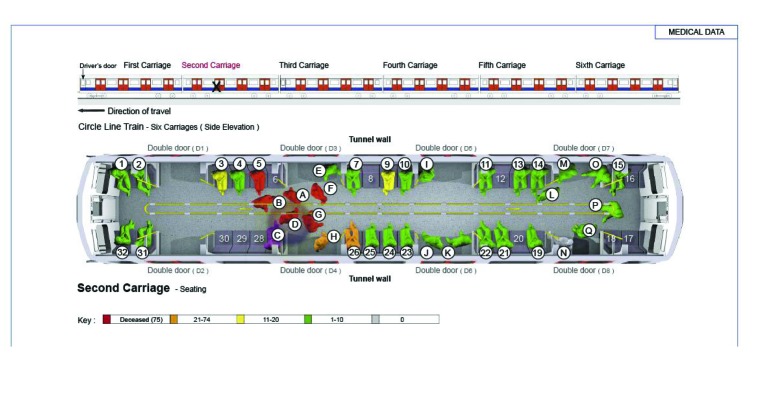
Pre-blast victim positional analysis at the Edgware Road scene

**Figure 3:**
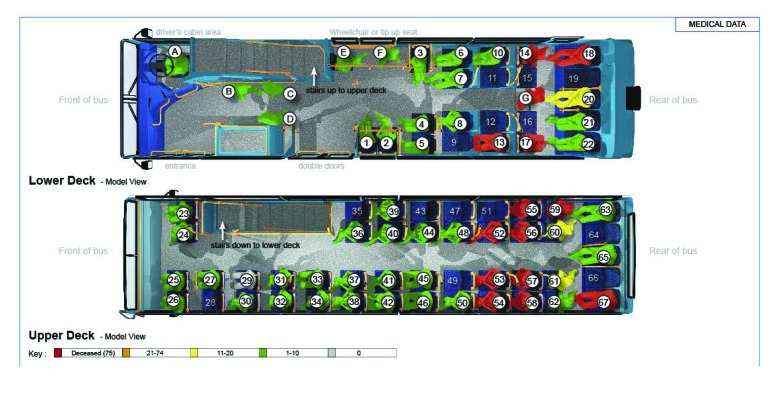
Pre-blast victim positional analysis at the Tavistock Square scene

**Figure 4:**
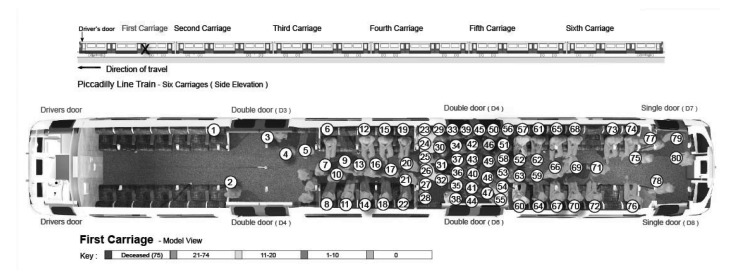
Pre-blast victim positional analysis at the King’s Cross scene. (At this scene only those present in the carriage reporting injuries were included in the analysis.)

Figures 1–4 highlight the locations of the donors and recipients of human projectiles at the four bombing sites as indicated on Table 2. At Aldgate, Edgware Road and Tavistock Square all those seated were given a number according to a seating plan and those standing were identified by letter.

**Table 2:** Identification of allogenic bone in victims of the London bombings

Bomb blast scene	Patient status	Body part embedded	Body part from	Recovered from
Aldgate	Died	Left maxilla	14	17
Aldgate	Survivor	Left foot	J	Unknown
Aldgate	Survivor*	Bone	Bomber	0
Aldgate	Survivor*	Bone	Bomber	0
Edgware Road	Survivor	Bone	Bomber	32
Tavistock Square	Died	Left toe	56	52
Tavistock Square	Survivor	Bone	Bomber	60
Tavistock Square	Survivor	Bone	Bomber	50
King’s Cross	Survivor	Bone	Bomber	20
King’s Cross	Survivor	Bone	Bomber	61
King’s Cross	Survivor	Bone	Bomber	23
King’s Cross	Survivor	Bone	Unknown	25

* Bone shards in the same survivor

Because of the tight packing at King’s Cross, it was not possible to use this method. Instead, each person was given a number and then positioned according to a multistep analysis of injuries. The first step was analysis of witness statements from survivors but this proved to be unreliable. It became clear from a number of ambiguities that some people entering underground carriages have little positional or spatial awareness, inaccurately positioning themselves and others around them. This was further compounded by the traumatic episode they suffered. The next two steps involved a review of specific injuries suffered by fatalities and survivors as well as charring patterns to the bodies of fatalities. Using these combined methods, maps were drawn up of position, distance from the devices and aspect of victims’ bodies towards the devices.

## Results

Most of the projectile fragments originated from the suicide bombers and some from the victims very close to the device. Of the 12 patients identified who had human projectile injuries, 8 originated from the bombers, 3 from the victims and 1 was unidentified (not subject to DNA profiling). Two patients died at the scene and the remaining ten were treated in hospital. Using DNA testing, the two patients who died at the scene had body parts embedded from other fatalities. Table 2 highlights the different injuries sustained in all four bombing scenes.

The identification of human tissue parts at King’s Cross had a very different pattern of distribution to that at both Aldgate and Edgware Road. This occurred because of the fireball effect of the bomb within a confined and crowded area of 5 persons per square metre. The dense crowd around the bomber absorbed most of the blast wave with no dissipation of blast energy due to the tight tunnel. As a result, there were four times the number of people killed at King’s Cross than at the other underground explosion sites. It can be assumed from photographic evidence and proven from computer trials that most of those who died were engulfed by the fireball although cause of death would have been multifactorial.

The situation on the bus at Tavistock Square was different again as there was collapse of the upper deck into the lower deck and there were too many projectiles to map them accurately. There were blast injuries for the upper deck and crush injuries in the lower deck due to collapse.

## Discussion

The blast wave from the detonation of a device is a high pressure (overpressure) shock wave that is then converted into a blast wind. The primary fragments are those originating from the bomber and the device. Secondary fragmentation comes from surrounding victims and other debris thrown out, resulting in penetrating wounds. An explosion in an underground train can lead to severe primary blast injuries as the blast wave is magnified by the walls of the tunnel and the structure of the carriage. The majority of the victims sustaining primary blast injuries will not survive. A vast majority of the injured who survive will have sustained secondary or tertiary injuries and this is dependent on the distance from the device. This is the situation for the survivors of the London bombings.

We report 12 cases of victims exposed to foreign body projectiles. It is possible that several more were affected but not identified due to the unique nature of these injuries and lack of awareness by medical staff. It is also believed that a number of fatalities not subject to hospital examination suffered impacted human bone fragments but as a result of external-only post-mortem examinations, the data are not available.

Two specific patterns of body parts were found outside the carriages at each scene. The first was the spread of parts at the point of detonation and is indicative of dynamic release. There was a second pattern of body parts around the trackside between the post-detonation carriage position and the carriage coming to rest. The authors believe that much of this second pattern is due to disturbance by rescuers and evacuees. Inside the carriage, the mapping results for body parts tended to be consistent with the positions of the victims compared with the device in the immediate vicinity (2m radius) of the explosion. Further afield, the location cannot be relied on to be accurate as there was substantial interference with the integrity of the scenes by rescuers and evacuees.

The missing body parts indicate the significant strength of the blast wave and this complicated the analysis. Furthermore, only body parts that could be positioned with a modicum of accuracy were plotted. In a number of cases, the system of photographing significant body parts in situ and logging the grid position was insufficient to give a reliable position.

A major drawback of the witness statements was the unreliability of recollection of the individual’s position relative to the device and to those around them. Commuters entering trains on the underground system are not particularly aware spatially and this is compounded by shock and confusion after the blast. This fault was highlighted very early in the plotting process, requiring a remedial and more accurate method of evaluation to be put in place. Due to the subjective nature of witness statements, especially during and after such an overwhelming life event, it was important to validate the individual positions. We validated these using a stepped process beginning with witness statements, then analysis of visible and non-visible injuries.

Our data from the three underground scenes on 7 July 2005 show that the number of injuries sustained from human projectiles is dependent on the position of the device, the bomber and the victims. However, the most relevant factor is the crowd density, where several victims who die will have sustained human projectile injuries. The survivors will be a further distance away and will have sustained human projectile injuries and survived

The train at King’s Cross showed that a bomb detonated in a densely packed crowd resulted in the generation of multiple biological secondary projectiles and subsequent enveloping within a fireball. Nevertheless, the impact of human projectile injuries was limited to a much smaller area due to crowd density. Furthermore, the position of the bomber at the time of detonation was different to that in the other two explosions. His position was more vertical and, as a result, the disruption to his body was limited, with the torso being intact but the injury affecting the front of the head and all limbs being amputated.

**Figure 5:**
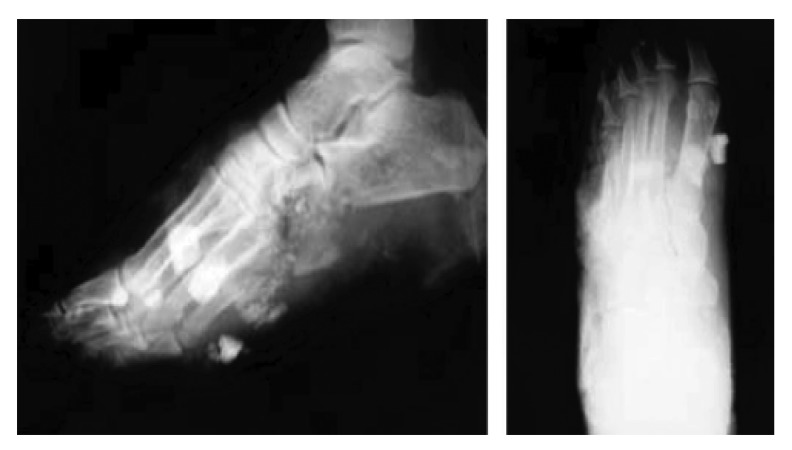
X-rays detailing multiple foreign bodies in left foot of a victim of the London bombings

During an explosion, any object can become a projectile (ie glass, debris etc). Due to its light weight and mechanical properties, the bone is a particularly effective projectile. Impact by non-human projectiles is treated as an open fracture while human projectile impacts, in addition to generating soft tissue injuries, raise the possibility of disseminating blood borne diseases. Both autogenic and allogenic fragments could be present in the same site (Fig 5). It is possible that in some cases suicide bombers may deliberately infect themselves with blood borne viruses in order to inflict maximum damage and in one suicide blast, bone fragments retrieved from a patient were reported to be positive for hepatitis B surface antigen.^7^ It is therefore essential to have all biological implanted foreign bodies tested for hepatitis B, hepatitis C and HIV as this has implications for victims and healthcare workers.

On 8 July 2005 the Health Protection Agency convened an expert group to advise on the management of these injuries. It recommended all patients with penetrating injuries or exposure to blood should be vaccinated against hepatitis B and serum taken to be stored for later testing should this become necessary.^8^ In addition, all recovered bone fragments or body parts must be given to the police for forensic testing. This should include DNA tests as well as testing for blood borne viruses. In the chaos that ensued in the aftermath of the bombings and because of ignorance of these issues, victims were not tested for blood borne viruses. In suicide bombings, the alleged bomber should, if possible, be tested for hepatitis B and C as well as HIV. This will help the medical carers to decide the best course of action for those involved.

In the London bombings it was too late to test for hepatitis B, hepatitis C or HIV. Looking back at the bombings, the lessons learnt should be included in all hospital protocols. Although one of our patients who was able to consent was commenced on antiretroviral therapy, the risk of HIV is so low as to not require action but patients need to be counselled and tested if necessary at the discretion of their medical carers.

The prevention of occupational infection by blood borne viruses relies on avoiding exposure and receiving immunisation and post-exposure prophylaxis. Preventive strategies are key because effective immunoprophylaxis is not likely to be available for hepatitis C or HIV in the near future. There must be accurate post-exposure follow-up for hepatitis C to eliminate or minimise the risk of transmission. Every effort should be made to reduce the risk of occupational exposure by introducing safer devices and techniques.

**Figure 6:**
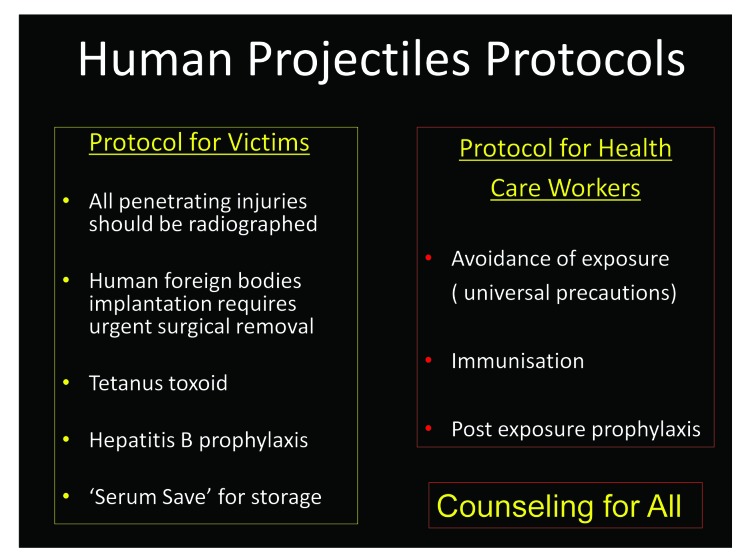
Protocols for human projectile injuries

In any emergency situation, healthcare providers and rescuers are also at risk from infection with blood borne pathogens, including hepatitis B virus, hepatitis C virus and HIV. Fears of blood borne infection transmission, especially HIV, have heightened awareness of the importance of occupational exposure as a source of these infections. The protocols put in place are shown in Figure 6. The data in the literature show that the risk of occupational percutaneous exposure to HIV from needles and other contaminated devices range from 0.2% to 0.5%.^9,10^ All the victims and healthcare workers should be counselled.

## Conclusions

Bomb blasts can cause the throwing out of high velocity projectiles and a spray of blood products, creating a risk of blood borne pathogens for the victims as well as emergency care and rescue workers. Every healthcare worker needs to be aware of human projectile injuries.
